# Protein with twin binding sites for uranium extraction from seawater

**DOI:** 10.1093/nsr/nwaf126

**Published:** 2025-03-29

**Authors:** Qisheng Zhou, Xuewen Cao, Jiacheng Zhang, Yan Li, Xinfeng Du, Yue Ma, Zhanhu Guo, Yihui Yuan, Ning Wang

**Affiliations:** State Key Laboratory of Marine Resource Utilization in South China Sea, Hainan University, China; State Key Laboratory of Marine Resource Utilization in South China Sea, Hainan University, China; State Key Laboratory of Marine Resource Utilization in South China Sea, Hainan University, China; State Key Laboratory of Marine Resource Utilization in South China Sea, Hainan University, China; State Key Laboratory of Marine Resource Utilization in South China Sea, Hainan University, China; State Key Laboratory of Marine Resource Utilization in South China Sea, Hainan University, China; Department of Mechanical and Civil Engineering, Faculty of Engineering and Environment, Northumbria University, UK; State Key Laboratory of Marine Resource Utilization in South China Sea, Hainan University, China; State Key Laboratory of Marine Resource Utilization in South China Sea, Hainan University, China

## Abstract

The ladder step-inspired uranyl binding protein fiber with twin uranyl binding sites achieves a record-high uranium extraction capacity of 25.6 mg g^−1^ from seawater in terms of adsorption methods.

Uranium is a key raw material for fabricating nuclear fuels, and the rapid growth of the nuclear energy industry has significantly increased the demand for a sustainable supply of uranium resource [1]. However, the uranium resources available in terrestrial exploitable ores are limited and can only support the nuclear energy industry for ∼60 to 80 years, without accounting for the rising consumption rates [2,3]. As a result, there is a pressing need to explore alternative uranium sources. In contrast, global seawater is estimated to contain around 4.5 billion tons of uranium, which is 1000 times greater than the uranium reserves found in terrestrial exploitable ores, and is expected to provide an almost inexhaustible supply of uranium [4]. Therefore, the extraction of uranium from seawater has garnered considerable attention in recent decades.

Among various methods for extracting uranium from seawater, adsorption has emerged as one of the most promising approaches, leading to the development of numerous adsorbents [5]. Notably, adsorbents based on biomacromolecules, such as protein and DNA, demonstrate superior advantages in terms of adsorption selectivity and binding affinity, enabling them to achieve remarkable uranium extraction capacities in natural seawater [6]. The exceptional uranium extraction performance of biomacromolecule-based adsorbents primarily results from their precise uranyl coordination structures, which are formed through the well-organized spatial arrangement of functional groups like carboxyl and amino groups [7].

However, maintaining these precise coordination structures requires the presence of auxiliary structures that do not directly contribute to uranyl binding, yet play a crucial role in preserving the protein's proper spatial conformation (Fig. 1a). Similar to many other metal-binding proteins, a single uranyl-binding protein typically contains only one specific binding site for the uranyl ion, which limits the potential for further enhancing the adsorption performance of protein-based uranium extraction materials in seawater [8].

**Figure 1.**
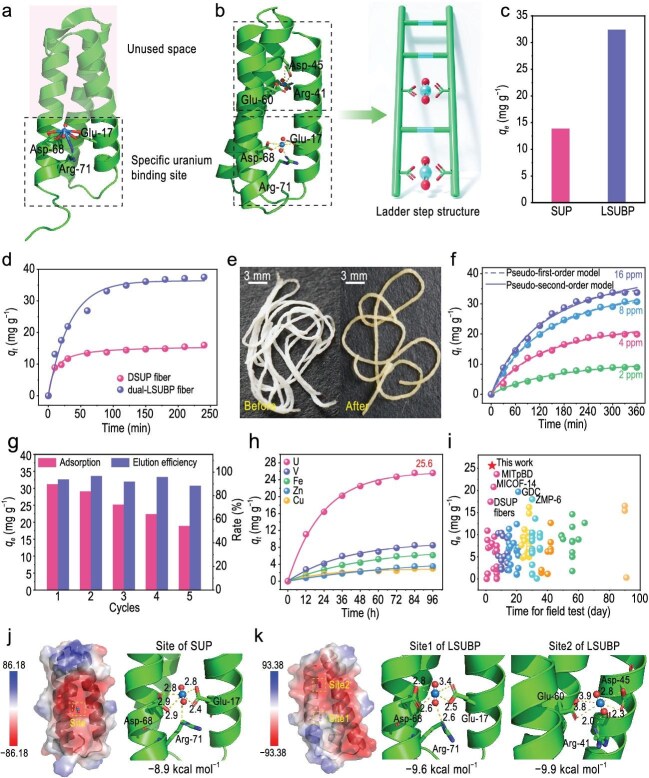
Structure and uranium adsorption performance of protein LSUBP, designed using a ladder-step-inspired strategy to incorporate twin uranyl binding sites. (a) Structure of protein SUP and the spatial utilization of its uranyl binding site. (b) Schematic of the ladder-step structure and the potential for incorporation of an additional uranyl binding site. (c) Uranium adsorption capacity of proteins LSUBP and SUP on a Ni-NTA resin column in 8 ppm uranium-spiked simulated seawater. (d) Adsorption kinetics of dual-LSUBP and DSUP fibers. (e) Morphology of the cross-linked dual-LSUBP hydrogel fiber before and after uranium adsorption. (f) Adsorption kinetics of the cross-linked dual-LSUBP fiber. (g) Reusability of the cross-linked dual-LSUBP fiber. (h) Uranium adsorption kinetics of the cross-linked dual-LSUBP fiber in natural seawater. (i) Uranium extraction capacities of different adsorbents in natural seawater, with corresponding references listed in the Supplementary Data. (j) Uranium adsorption mechanism calculations for protein SUP. (k) Uranium adsorption mechanism calculations for protein LSUBP.

Previously, a super uranyl-binding protein (SUP) with high affinity and selectivity for uranyl ions was designed, and the adsorbent based on SUP achieved a breakthrough uranium extraction capacity of 17.45 mg g^−1^ in natural seawater, demonstrating the high potential of this protein for uranium extraction from seawater [9]. However, the capacity for further improvement is limited due to the presence of only a single-uranyl-ion-binding site in one SUP protein. Structural analysis of SUP revealed that it consists of three α-helices arranged nearly in parallel, forming a supporting framework similar to a ladder structure, with the uranyl binding site located among these three α-helices like a step on a ladder (Fig. 1b). Given that the three α-helices form closely packed internal cavities in an almost parallel orientation, introducing twin uranyl binding sites in a ladder-step-like fashion within the three α-helices could enhance the uranium adsorption capacity of the protein-based material.

In this study, an additional uranyl binding site was introduced by mutating Val45 to Asp45 and Ile60 to Glu60 in SUP, which exhibited the highest uranium adsorption capacity among all tested mutants (Fig. S1 and Table S1). The mutant amino acid residues, Asp45 and Glu60, provide carboxyl oxygen atoms that could form a coordination site for the uranyl ion. Additionally, the hydrogen atom of the amino acid residue Arg41 may form a hydrogen bond with the oxygen atom from the uranyl ion. This newly incorporated uranyl binding site can act as a twin of the original uranyl binding site in SUP to enhance the uranium adsorption capacity. Structural superposition analysis between the structures of the SUP protein and the mutant protein yielded an root mean square deviation (RMSD) value of 0.329 Å, suggesting that the mutation did not significantly alter the protein's overall conformation (Fig. S2). The mutant protein was named ladder-step-inspired uranyl binding protein (LSUBP) because of the incorporation of the uranyl binding site through the ladder-step-inspired strategy. It was successfully expressed in *Escherichia coli* strain BL-21, yielding a protein with a molecular weight of ∼15 kDa (Fig. S3). Far-ultraviolet circular dichroism (UV-CD) spectroscopy analysis revealed that the secondary structure of LSUBP was primarily composed of α-helices, which aligned with the predicted protein structure and indicated that the protein maintained a suitable spatial configuration for binding uranyl ions (Fig. S4).

To verify the uranium adsorption ability of the constructed mutant, the purified LSUBP protein was loaded onto a nickel-nitrilotriacetic acid (Ni-NTA) resin column, and its uranium adsorption capacity was tested using 8 ppm uranium-spiked simulated seawater passed through the resin. The results showed that the LSUBP protein achieved a high uranium adsorption capacity of 31.76 mg g^−1^, which is 2.31 times that of SUP (Fig. 1c). To further evaluate its potential for use in fabricating uranium adsorbents, we constructed a chimeric protein dual-LSUBP consisting of two identical amino acid sequences of LSUBP. Protein hydrogel fibers, called dual-LSUBP fibers, were then fabricated using biomimetic spinning technology [10]. The dual-LSUBP fibers exhibited

a high uranium adsorption capacity of 37.53 mg g^−1^ in 8 ppm uranium-spiked simulated seawater, which is 2.34 times that of the dual-SUP (DSUP) fibers, confirming that the strategy successfully incorporated twin uranyl binding sites in a ladder-step fashion (Fig. 1d).

To improve the strength of protein hydrogel fiber for use in natural seawater, the protein hydrogel fiber underwent a cross-linking process via soaking in a 1% glutaraldehyde solution for 15 minutes at 25°C. The cross-linked dual-LSUBP fiber formed a hydrogel and appeared white in its hydrated state, with a diameter of ∼480 nm. Upon uranium binding, the color changed to yellow (Fig. 1e). The cross-linked dual-LSUBP fiber showed the highest uranium adsorption capacity at pH 5.0 and the increase in temperature can benefit the adsorption performance (Figs S5 and S6). The adsorption kinetics analysis demonstrated that the cross-linked dual-LSUBP fiber achieved a uranium adsorption capacity of 30.70 mg g^−1^ and 33.75 mg g^−1^ in 8 ppm and 16 ppm uranium-spiked simulated seawater within 4.5 hours (Fig. 1f). The adsorption behavior closely followed the pseudo-second-order model, suggesting a chemical adsorption mechanism. Adsorption isotherm analysis indicated that the maximum theoretical uranium adsorption capacity of the cross-linked dual-LSUBP fiber was 39.80 mg g^−1^ (Fig. S7 and Table S2). Notably, despite the incorporation of an additional uranyl binding site in LSUBP, the cross-linked dual-LSUBP fiber maintained high selectivity against commonly occurring interfering ions in seawater, indicating that the new binding site displayed a strong preference for uranyl ions (Figs S8 and S9). Due to the cross-linking process, the cross-linked dual-LSUBP fiber exhibited high reusability, maintaining a uranium adsorption capacity of 18.80 mg g^−1^ after five reuse cycles, which was higher than the adsorption capacity of the newly fabricated DSUP fiber (Fig. 1g). X-ray photoelectron spectroscopy (XPS) and energy-dispersive X-ray spectroscopy (EDS) analyses confirmed the uniform adsorption of uranium by the cross-linked dual-LSUBP fiber in the state of uranyl ions (Figs S10–S12).

To assess the practical uranium extraction capability in natural seawater, the cross-linked dual-LSUBP fiber was placed in a column flow system with filtered seawater flowing through the column at a rate of 1 L min^−1^. The results revealed that the cross-linked dual-LSUBP fiber exhibited an exceptionally high uranium adsorption capacity of 25.60 mg g^−1^ with an equilibrium adsorption time of 4 days, representing a 46.7% improvement compared to the DSUP fiber (Fig. 1h). This performance outperforms all previously reported seawater uranium extraction materials based on the adsorption principle (Fig. 1i; Table S3). Furthermore, the cross-linked dual-LSUBP fiber maintained high adsorption selectivity in natural seawater, with an adsorption ratio of 3.02 for uranium to vanadium [9]. These findings suggest that the cross-linked dual-LSUBP fiber has strong potential for use in uranium extraction from natural seawater.

To further elucidate the adsorption mechanism, molecular docking was used to analyze the coordination interactions between the incorporated uranyl binding site and the uranyl ion. The results showed that both the initial uranyl binding site from SUP and the newly incorporated uranyl binding site exhibited high binding energies with the uranyl ion, contributing to the high uranium adsorption performance of LSUBP (Fig. 1j and k). In addition to the uranyl binding site derived from SUP, the newly incorporated binding site adsorbed the uranyl ion through a coordination structure formed between the oxygen atoms of Asp45 and Glu60 and the uranium atom of the uranyl ion, as well as hydrogen bonds between the hydrogen atom of Arg41 and the oxygen atom of the uranyl ion, which together stabilize the uranyl ion binding.

The design of high-performance uranium adsorbents is crucial for the effective extraction of uranium resources in seawater. This study introduces a ladder-step-inspired strategy for constructing uranyl-binding proteins with twin uranyl binding sites to enhance uranium extraction performance. Due to the incorporation of additional uranyl-binding sites, the fiber produced using LSUBP exhibited a 46.7% improvement in uranium extraction capacity in natural seawater, compared to the protein fiber made with protein SUP. This performance surpasses that of all other seawater uranium extraction materials based on adsorption principles. The findings of this study provide a promising new candidate for uranium extraction from seawater and offer valuable insights for the design of adsorption materials, with the aim of improving the adsorption capacity of metal ions.

## Supplementary Material

nwaf126_Supplemental_File
