# Supplementary material for: Protein with twin binding sites for uranium extraction from seawater
Source: Natl Sci Rev. 2025 Mar 29;12(5):nwaf126. doi: 10.1093/nsr/nwaf126 (PMC12060868; doi:10.1093/nsr/nwaf126)
Supplement: nwaf126_Supplemental_File [file nwaf126_supplemental_file.pdf]

Supplementary Information

Protein with twin binding sites for uranium extraction from seawater

Qisheng Zhou[1,†], Xuewen Cao[1,†], Jiacheng Zhang[1], Yan Li[1], Xinfeng Du[1], Yue Ma[1], Zhanhu Guo[2], Yihui Yuan[1,*], and Ning Wang[1,*]

[1]State Key Laboratory of Marine Resource Utilization in South China Sea, Hainan University, Haikou 570228, China

[2]Department of Mechanical and Civil Engineering, Faculty of Engineering and Environment, Northumbria University, Newcastle Upon Tyne NE1 8ST, UK

*Corresponding authors. E-mails: wangn02@foxmail.com, yuanyh@hainanu.edu.cn

Methods

Design and preparation of the protein mutant

To enhance the adsorption capacity of recombinant protein fiber, gene mutation technology was used to mutate the available space outside the uranium-binding site of the protein SUP. Homology modeling was conducted using SWISS-MODEL to ensure that the protein's spatial structure did not undergo significant changes. The gene for the mutant was inserted into the NdeI/XhoI sites of the expression vector pET-22b (+) and subsequently transferred into *Escherichia coli* strain BL-21 (DE3) to construct recombinant strains for fusion protein expression.

The engineered strains were cultured in LB medium containing 10 mg L^{-1} ampicillin (10 g L^{-1} NaCl, 5 g L^{-1} yeast extract, 10 g L^{-1} tryptone) and incubated overnight at 37 ℃ with shaking at 180 rpm. Then, the bacterial culture was inoculated at a 1:100 ratio into fresh LB broth and cultured for an additional 3-4 hours until the optical density at 600 nm reached 0.8. IPTG was added to the culture to induce fusion protein expression overnight at 20 ℃. The bacterial cells were subsequently harvested by centrifugation at 10,000 rpm for 15 minutes at 4 ℃. The cells were lysed using a high-pressure homogenizer, and the supernatant containing the recombinant protein was collected after centrifugation at 10,000 rpm for 30 minutes at 4 ℃. Recombinant proteins were purified using a Ni-NTA resin column, and the purified proteins were analyzed by SDS-PAGE. 0.5 mg of the purified protein was loaded onto a Ni-NTA resin column, and 50 mL of 8 ppm uranium-spiked simulated seawater was flow through the resin to determine the adsorption performance.

Biomimetic spinning of the protein hydrogel fiber

The designed SUP mutant was used to construct a fusion protein. Two gene fragments of the protein mutant were ligated using a linker (NH_2-GGGGGSGGGGSGGGGS-COOH) to form the recombinant gene of dual-LSUBP. The codon-optimized gene of dual-LSUBP gene was then inserted into *Escherichia coli* strain BL-21 (DE3), and the recombinant protein dual-LSUBP was expressed and purified using the same method as described earlier. dual-LSUBP fibers were obtained via biomimetic spinning. The spinning solution used in this study

consisted of 100 mM sodium phosphate (pH > 5.5), 100 mM sodium acetate (pH 4.0-5.5), and 200 mM citric acid (pH < 4.0). After spinning, the protein fibers were crosslinked in a 1% glutaraldehyde solution for 15 minutes to enhance their stability.

Characterization of the recombinant protein and protein fiber

SDS-PAGE was used to analyze the molecular weight of the purified mutant protein. Far-ultraviolet circular dichroism (CD) was employed to determine the secondary structure of the protein, with the data were analyzed using the software CDNN. The microstructure of the protein fibers was visualized using a Thermo Scientific Verios G4 scanning electron microscope (SEM) and energy dispersive spectroscopy (EDS) was conducted. X-ray photoelectron spectroscopy (XPS) analysis was carried out using a Thermo Scientific ESCALAB 250Xi spectrometer. Inductively coupled plasma mass spectrometry (ICP-MS) was used to determine the concentration of uranium and other low-concentration competing ions in seawater.

Uranium adsorption kinetics analysis

Protein fibers with a dry weight of 5 mg were immersed in 500 mL of 8 ppm uranium-spiked simulated seawater at pH 5.0 to determine their uranium adsorption capacity. Every 30 minutes, aliquots of the solution were collected, and the uranium concentration was measured using a UV-Vis spectrophotometer. The uranium adsorption capacity was calculated using the following formula (1):

$$q_t = \frac{(C_0 - C_t)\times V}{m} \quad (1)$$

where t represents the contact time; q_t (mg g^{-1}) is the uranium adsorbed after time t; C_0 (mg L^{-1}) is the initial uranium concentration; C_t (mg L^{-1}) is the uranium concentration at time t; V (L) is the volume of the uranium solution; m (g) is the mass of the adsorbent.

Reusability analysis

To evaluate the reusability of the cross-linked dual-LSUBP fibers, 5 mg fibers were firstly immersed into 500 mL uranium-spiked simulated seawater until equilibrium adsorption was achieved. Then uranium bound to the fibers was eluted with 100 mM EDTA at pH 8.0 for 30 min. The uranium elution rate and the

reusability of dual-LSUBP fibers were determined using the formula (1). After each elution, the protein fibers were washed three times with deionized water for reuse in the next adsorption-desorption cycle.

Adsorption selectivity analysis

To assess the selectivity of the dual-LSUBP fiber for marine metal ions, nine metal ions (UO_2^{2+}, VO^{3-}, Fe^{3+}, Co^{2+}, Ni^{2+}, Cu^{2+}, Zn^{2+},Mn^{2+}, Sr^{2+}) were selected and added to natural seawater to achieve a final concentration 100 times that of the ion in natural seawater. Other major ions (Na^+, K^+, Ca^{2+}, Mg^{2+}) were maintained at their original concentrations in natural seawater. Subsequently, protein fiber with dry weight of 5 mg was added to 500 mL of the mixed solution and allowed to adsorb for 1 hour. The concentration of each metal ion in the solution was measured using ICP-MS.

Uranium extraction ability in natural seawater

To evaluate the uranium extraction capacity of dual-LSUBP fibers in natural seawater, the fiber was placed in a column flow system. Natural seawater was filtered through a 0.22 μm membrane to eliminate microorganisms and particulate matter. In each experiment, 100 L of seawater and protein fibers with dry weight of 10 mg were used, and seawater was passed through the column at a flow rate of 1 L min^{-1}. At 25 ℃, the uranium concentration in the seawater was measured every 12 hours using ICP-MS.

Structural superposition of SUP protein and LSUBP protein

Based on the protein structure files, PyMOL software was used to perform structural alignment, employing the protein backbone atoms as reference points. The alignment was executed using the “align” function within PyMOL, from which the RMSD values were calculated. Subsequently, the structurally variable amino acid residues were labeled and comparative local structural images were generated to visually illustrate the detailed structural differences before and after mutation.

Simulation of the coordination structure by molecular docking

First, the protein structure was processed using UCSF chimera [1],

AMBER14SB atomic charges were assigned, and was calculated using the H + + 3 online tool [2, 3]. The ligand uranyl ion structure generated 3D structures through the open-source chemoinformatics software package RDKit [4], and performed conformational sampling, optimized configurations using the MMFF94 force field, and output low-energy conformations, assigning AM 1-BCC local charges using UCSF Chimera. In this study, the molecular docking experiment was performed used AutoDock4.2 software [5], with the ligand as a whole, the box size was set to the cube with side length 22.5Å, and the Spacing step was set to 0.375. The docking box center of different proteins is set according to the site specified by the customer, and the maximum limit number of search conformations is set to 10000. The conformation sampling and scoring are performed using the genetic algorithm.

Supplementary Figures

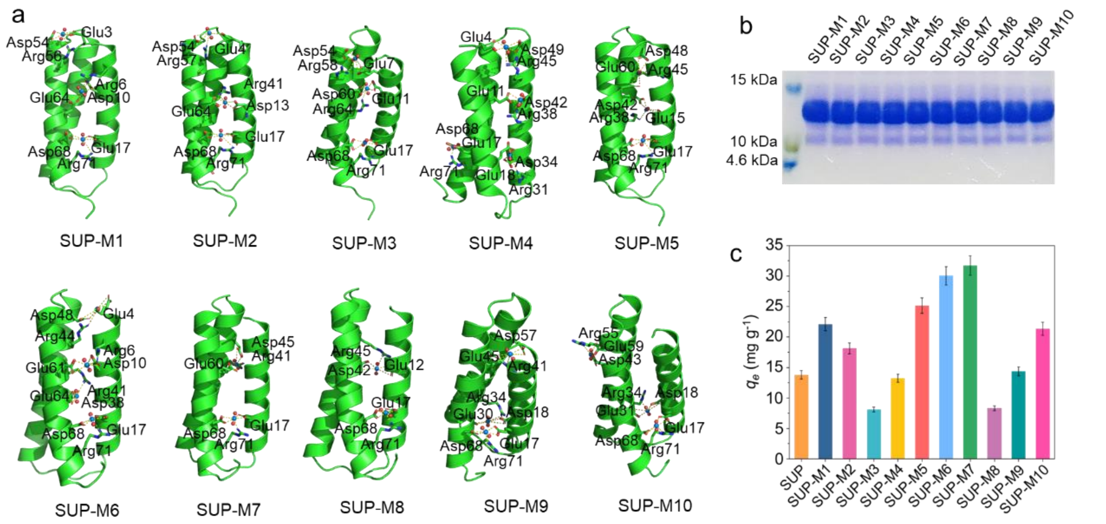

Figure S1. (a) Ten SUP mutants with additional uranyl binding sites. (b) SDS-PAGE electrophoresis results of purified mutant proteins. (c) Uranium adsorption performance of the purified mutant proteins loading on Ni-NTA column.

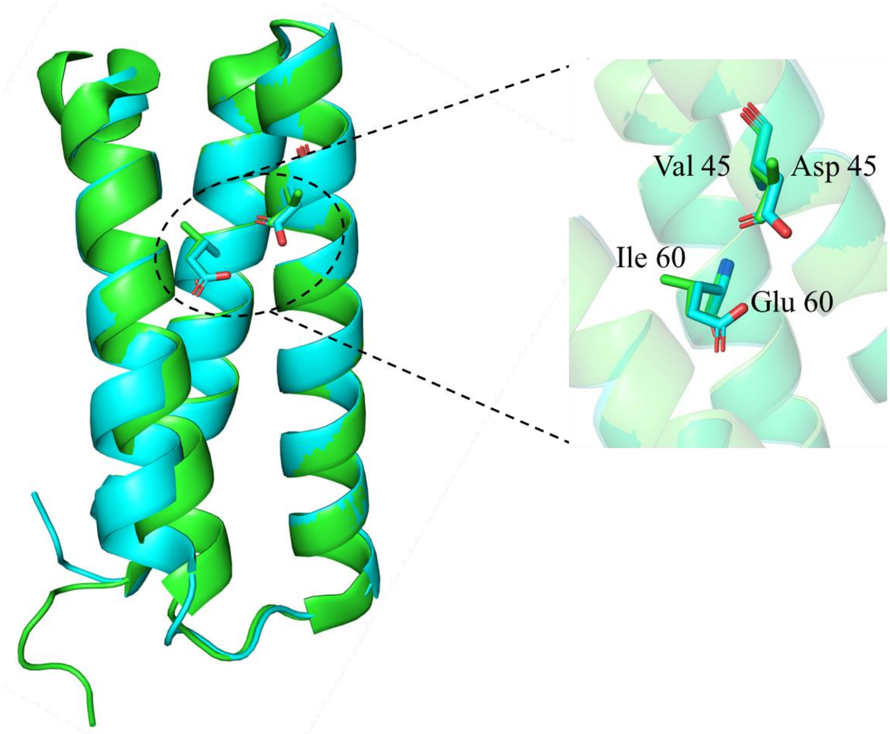

Figure S2. Structural superposition of the SUP protein and the mutant LSUBP protein.

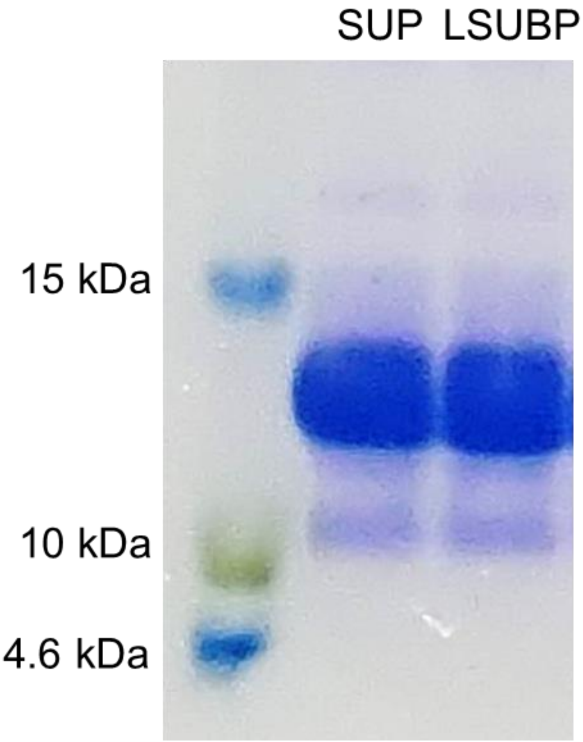

Figure S3. SDS-PAGE profiles of the purified SUP and LSUBP.

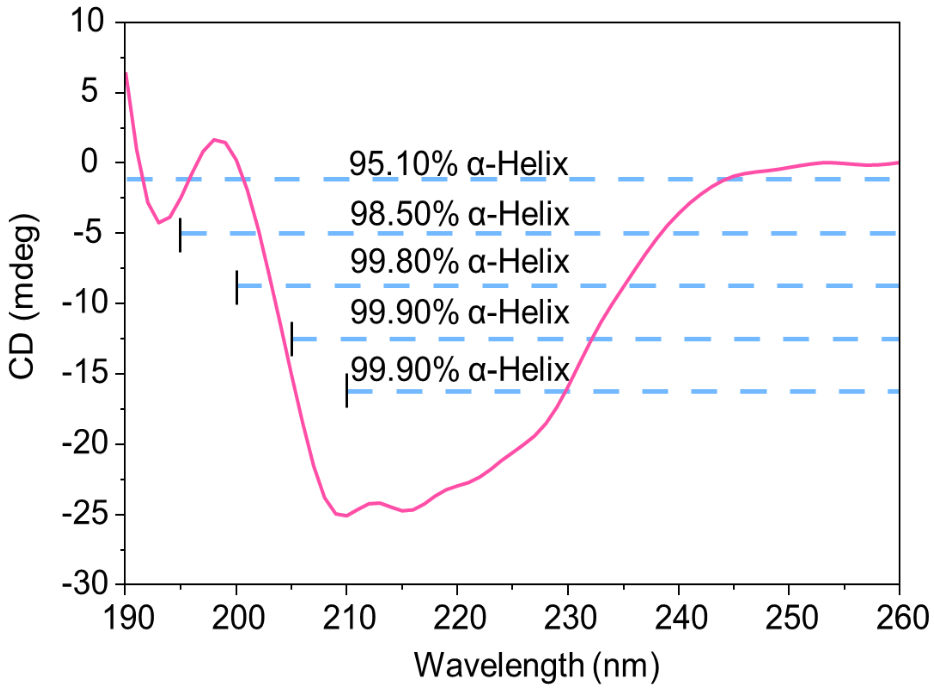

Figure S4. CD spectrum of LSUBP.

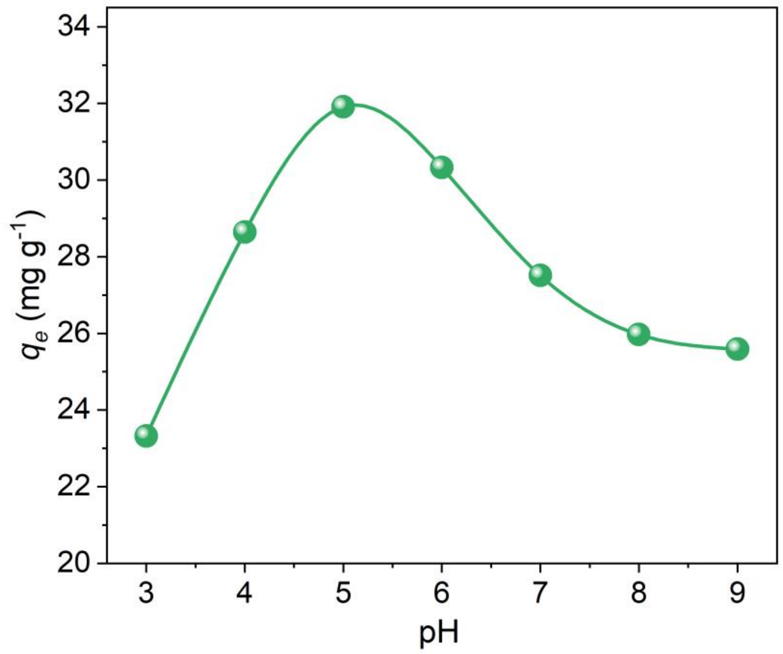

Figure S5. The effect of pH values on uranium adsorption capacity of the cross-linked dual-LSUPB fiber.

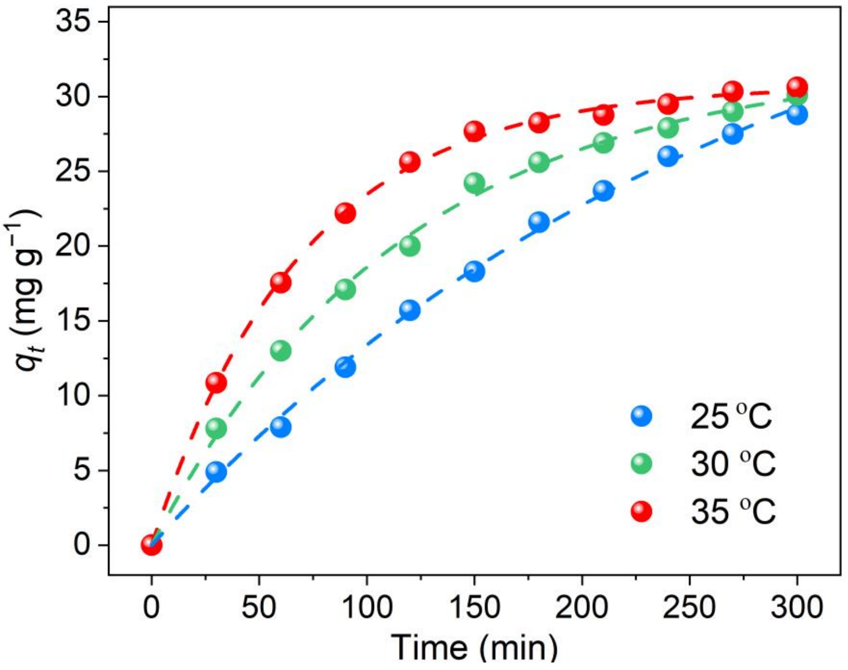

Figure S6. The effect of different temperatures on adsorption kinetics of the cross-linked dual-LSUPB fiber.

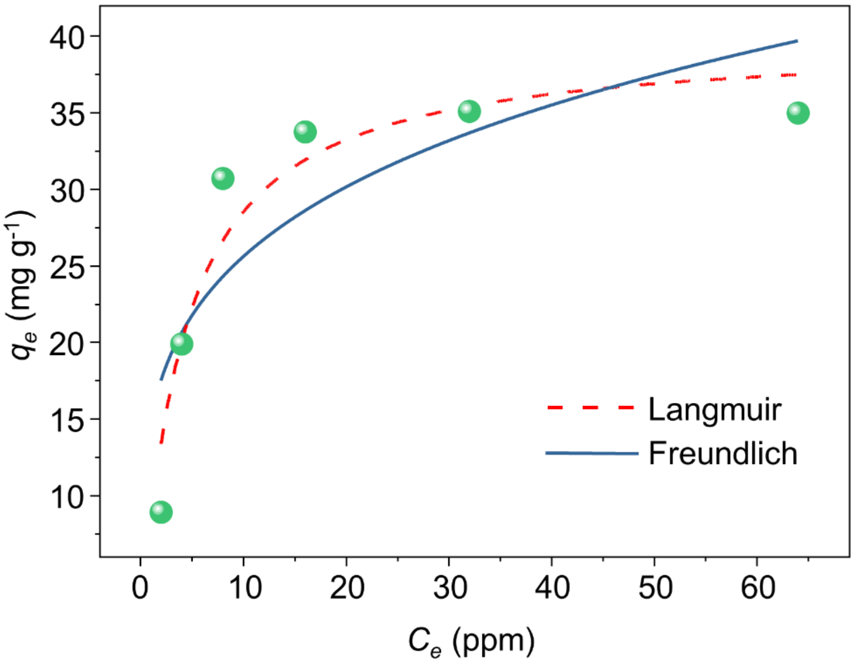

Figure S7. Equilibrium adsorption isotherms and the fitting result of the cross-linked dual-LSUPB fiber.

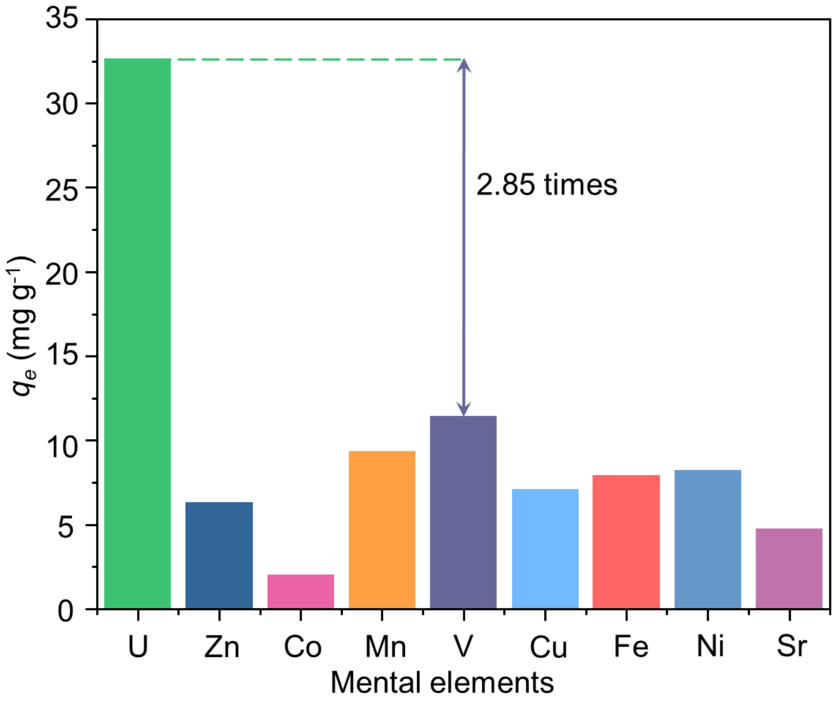

Figure S8. Selectivity of uranium adsorption of cross-linked dual-LSUPB fiber in 100 times spiked natural seawater.

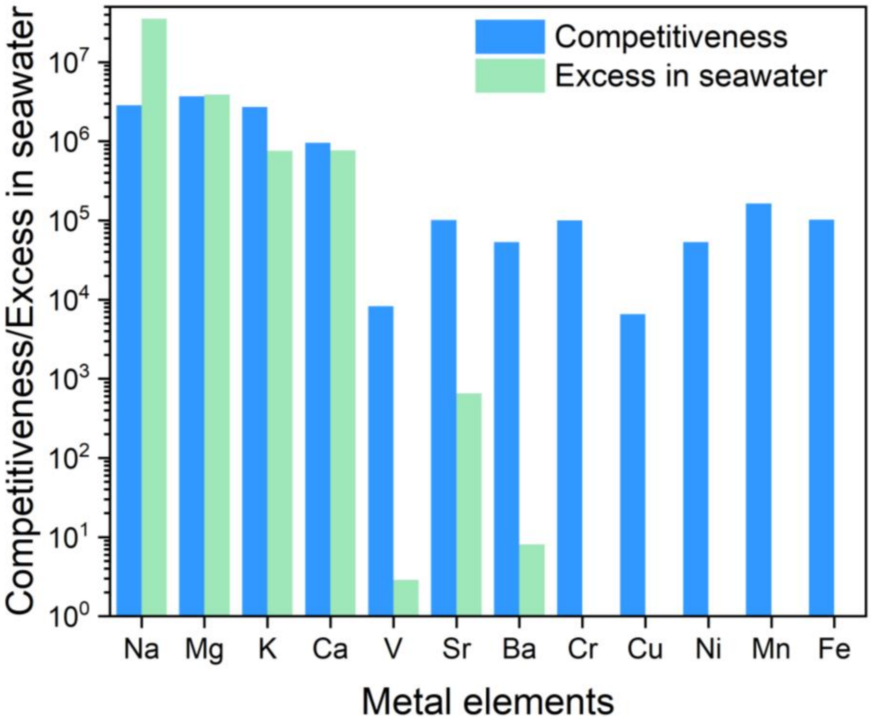

Figure S9. Competitive adsorption of the cross-linked dual-LSUBP fiber for uranyl ions over various other relevant metal ions.

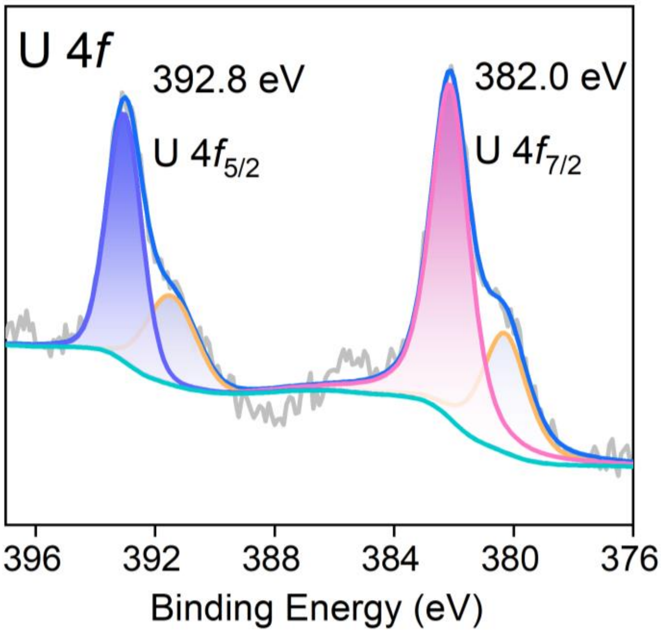

Figure S10. High-resolution XPS spectra of U 4*f* of the cross-linked dual-LSUBP fiber after uranium adsorption.

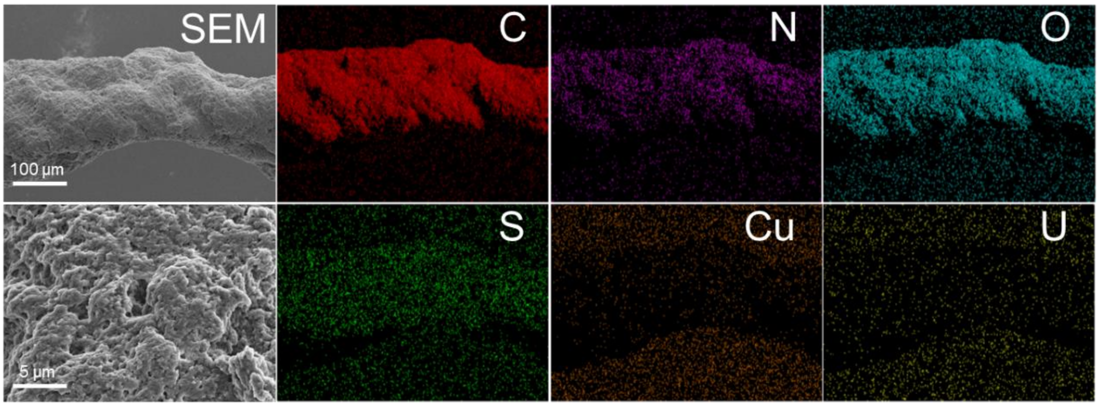

Figure S11. SEM and corresponding EDS images of the cross-linked dual-LSUBP fiber before uranium adsorption.

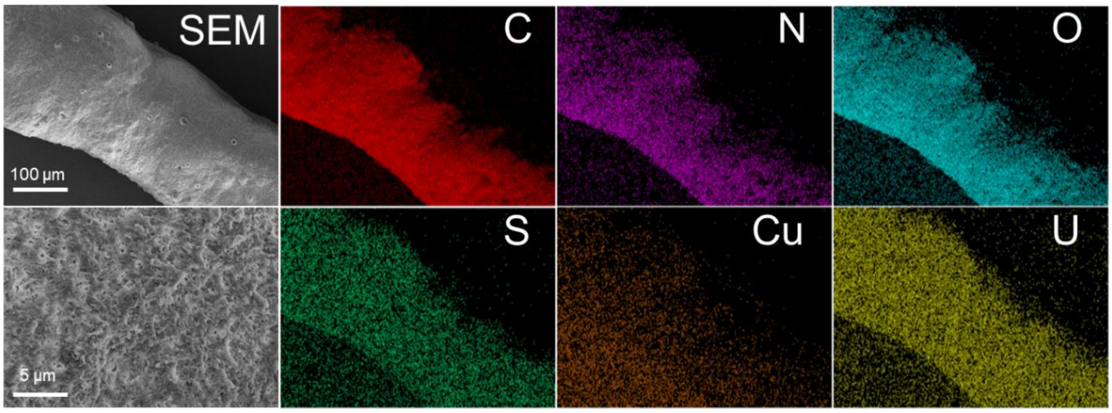

Figure S12. SEM and corresponding EDS images of the cross-linked dual-LSUBP fiber after uranium adsorption.

Supplementary Tables

Table S1. Characters and uranium adsorption of the corresponding material derived from protein SUP and LSUBP.

Name	Sequence	Binding site	Uranium adsorption capacity in natural seawater (mg g^{-1})
SUP	LDCRERIEKDLENLEKELME<br>MKSIKLSDDEEAVVERALNY<br>RDDSVYYLEKGDHITSFGCI<br>TYAQGLLDSLRMLHRIIEG	Site:<br>Glu17<br>Asp68<br>Arg71	17.45
LSUBP	LDCRERIEKDLENLEKELME<br>MKSIKLSDDEEAVVERALNY<br>RDDSDYYLEKGDHITSFGCE<br>TYAQGLLDSLRMLHRIIEG	Site1:<br>Glu17<br>Asp68<br>Arg71<br>Site2:<br>Glu60<br>Asp45<br>Arg41	25.60

Table S2. The fitting results of adsorption isotherm fitted by Langmuir and Freundlich models for uranium on the cross-linked dual-LSUBP fiber.

Langmuir			Freundlich		
q_{mL} (mg g^{-1})	K_L (L mg^{-1})	R^2	K_F ((mg g^{-1}) (L mg^{-1})$^{1/n}$)	$1/n_F$	R^2
39.80	0.25	0.9188	14.86	0.24	0.7071

Table S3. Summary of adsorbents used for uranium extraction from natural seawater.

Adsorbent	Time for field test（day）	Uranium extraction capacity（mg g^{-1}）	Rate for uranium（mg g^{-1} day^{-1}）	Reference	Year
LSUBP fiber	4	25.60	6.4	This work	
DSUP-OP	4	8.05	2.01	[6]	2024
CMPA-F/CFM	7	1.25	0.18	[7]	2024
MICOF-14	5	20.8	4.16	[8]	2024
TI-COF	1	8.8	8.8	[9]	2024
CS-AO-AMP	7	0.27	0.04	[10]	2024
MITpBD	7	23.66	3.38	[11]	2024
β-CD-g-PAO	1	10.9	10.9	[12]	2024
$SSPG_4$ film	10	10.4	1.04	[13]	2024
PGP hydrogel	11	9.73	0.88	[14]	2024
PAO-Y	15	1.97	0.13	[15]	2024
PAOM	14	2.03	0.15	[16]	2024
SA/PEI	14	3.58	0.26	[17]	2024
H-UiO-66-PA	15	6.76	0.45	[18]	2024
PAF-174-AO	20	12.4	0.62	[19]	2024
PN-300 nanotubes	15	7.01	0.47	[20]	2024
H-PDA/SA-ZIF-8	15	6.9	0.46	[21]	2024
P5-AO	18	8.1	0.45	[22]	2024
A-PAA@WMPAO	15	4.79	0.32	[23]	2024
HFAO-QPEI	21	2.45	0.12	[24]	2024
LAM	20	0.99	0.05	[25]	2024
CPMA-AO	23	7.63	0.33	[26]	2024
Gel/PAM(DN)-HA	24	4.8	0.2	[27]	2024
PAO/PMPC-SH	28	6.26	0.22	[28]	2024
ZW-PAO hydrogel	25	9.38	0.38	[29]	2024
SF-g-PAO	28	4.95	0.18	[30]	2025
ZMP_2-6	30	18	0.6	[31]	2024
PE－PAO@ADH	30	6.76	0.23	[32]	2024
AO-HAP	30	6.25	0.21	[33]	2024
GO/g-C3N4/PAO	30	10.39	0.35	[34]	2024
PAO-CNF supramolecular composite hydrogels	35	6.6	0.19	[35]	2024
T-AO-HMO	35	9.67	0.28	[36]	2024
CS-MPC	42	8.28	0.2	[37]	2024
PA-bPEI	50	9	0.18	[38]	2024
CMCS/P(AO-co-AM)-Cu	56	14.62	0.26	[39]	2024

UUS-1/CdS	1.5	7.03	4.69	[40]	2023
COF 2-Ru-AO	3	7.36	2.45	[41]	2023
c-PVA-g-PAO&MXene NFs	7	2.23	0.32	[42]	2023
PM membrane	7	2.19	0.31	[43]	2023
PAO-BS	10	2.7	0.27	[44]	2023
Anti-GO-BPG-GLACS hydrogel	14	9.18	0.66	[45]	2023
AFNH	14	5.93	0.42	[46]	2023
COF-R_5	15	11.3	0.75	[47]	2023
ZP-PAN fibers	15	8.1	0.54	[48]	2023
PAO/PEI	15	9.79	0.65	[49]	2023
AO-PAF	16	3.22	0.2	[50]	2023
AO-PAM/Alg hydrogel	20	6.23	0.31	[51]	2023
PVDF-4-h	20	6.61	0.33	[52]	2023
MSF@PAO-PEI	20	1.1	0.06	[53]	2023
SPI hydrogel	21	5.29	0.25	[54]	2023
I-CNF aerogel	24	9.46	0.39	[55]	2023
ZIF-8-CN	28	16.2	0.58	[56]	2023
AOPEI-C-PAN fibers	28	9.54	0.34	[57]	2023
ECP gels	28	10.43	0.37	[58]	2023
Cu SA@UiO-66-NH_2	28	9.16	0.33	[59]	2023
^{CSMCRI}HOF2_P	28	14.8	0.53	[60]	2023
CPAO/CN	30	5.9	0.2	[61]	2023
TTh-COF-AO	30	10.24	0.34	[62]	2023
PAGM-1 hydrogel	30	6.1	0.2	[63]	2023
iMOF-1A	30	9.42	0.31	[64]	2023
DDH-PAO gels	30	6.3	0.21	[65]	2023
ZIF-67@SiO_2-A/PAM	30	6.33	0.21	[66]	2023
PAOBS-4	30	1.03	0.03	[67]	2023
AHCAOBS-3	30	1.19	0.04	[68]	2023
sy-PEI–PAA–PAO	30	10.45	0.35	[69]	2023
h-PAO hydrogel	49	9.86	0.2	[70]	2023
KIST hydrogel	1.17	1.17	1.17	[71]	2022
MGH	7	7.51	1.07	[72]	2022
m$CaCO_3$	7	6.5	0.93	[73]	2022
CS-AMP	7	0.04	0.01	[74]	2022
MUU_{re}	16	7.35	0.46	[75]	2022
AO-PIM-1	28	9.03	0.32	[76]	2022
PAO@CHM	28	7.46	0.27	[77]	2022
HA-PAO NFMs	28	6.73	0.24	[78]	2022
CI-PAO	28	6.17	0.22	[79]	2022
MP-PAO	28	5.8	0.21	[80]	2022
CMPM	30	5.81	0.19	[81]	2022

FF-PT	30	3.22	0.11	[82]	2022
VA-PG	32	13.63	0.43	[83]	2022
Fe_3O_4@TiO_2-AO	33	0.09	0.003	[84]	2022
Crab carapace of *P. trituberculatus*	36	1.38	0.04	[85]	2022
W-UiO	40	6.2	0.16	[86]	2022
Fe@PDA-PAO	42	12.67	0.3	[87]	2022
PAO-Co	49	9.7	0.2	[88]	2022
LDPE	56	1.79	0.03	[89]	2022
PAO-BSPE	64	12.67	0.2	[90]	2022
PAN-NH_2-AO	91	0.31	0.003	[91]	2022
Fe-N_x-C-R	1	1.2	1.2	[92]	2021
AO/g-C_3N_4	5.5	9.55	1.74	[93]	2021
DC-PAO	7	6.42	0.92	[94]	2021
ZIF-90-ABOA	7	2.8	0.4	[95]	2021
Tp-DBD	8	10.31	1.29	[96]	2021
UiO66-NH-(AO)	8	5.2	0.65	[97]	2021
PA-PAO/CS NFs	10	4.91	0.49	[98]	2021
CPP	10	4.15	0.42	[99]	2021
PLMR	10	2.14	0.21	[100]	2021
BHMS3	12	5.14	0.43	[101]	2021
GDC	21	19.7	0.94	[102]	2021
PPH-OP	21	7.12	0.34	[103]	2021
AOP@ZIF-8/TA	25	11.17	0.45	[104]	2021
AUPM	25	8.78	0.35	[105]	2021
NC-PAO DN hydrogel	25	8.62	0.34	[106]	2021
COF-HHTF-AO	25	5.12	0.2	[107]	2021
AF Anti-COF	28	6.64	0.24	[108]	2021
UiO-66-NH2@CS-PDA	28	5.52	0.2	[109]	2021
PAO-PHMB-A	30	3.19	0.11	[110]	2021
AOBS-M	30	0.97	0.03	[111]	2021
BP@CNF-MOF	42	6.77	0.16	[112]	2021
PAO-CB	56	8.56	0.15	[113]	2021
POP_1-AO	56	8.4	0.15	[114]	2021
Amidoximated AF1	56	5.04	0.09	[115]	2021
POP2-PO_3H_2	56	5.01	0.09	[116]	2021
GCZ8A	0.5	0.01	0.02	[117]	2020
DSUP fibers	3	17.45	5.82	[118]	2020
CMPs	5	2.3	0.46	[119]	2020
DNA–UEH	6	6.06	1.01	[120]	2020
NDA-TN-AO	27	6.07	0.22	[121]	2020
Zn^{2+}-PAO	28	9.23	0.33	[122]	2020
UiO-66-3C4N	28	6.85	0.24	[123]	2020

Anti-PAO gels	30	9.29	0.31	[124]	2020
CMC-PAM/$Mg(OH)_2$	30	8.6	0.29	[125]	2020
AP_{15} fibers	35	10.31	0.29	[126]	2020
PAO PNMs	35	9.35	0.27	[127]	2020
ZIF-67/$SAP_{0.45}$	35	6.99	0.2	[128]	2020
AOPAN/ZIF fibers	36	2.03	0.06	[129]	2020
PAO-GA	40	1.14	0.03	[130]	2020
CP-PAO hydrogel	42	6.21	0.15	[131]	2020
BP-PAO	56	11.76	0.21	[132]	2020
PAO/ Alg NFs	56	8.42	0.15	[133]	2020
LCW	56	6.02	0.11	[134]	2020
PAF-CS	60	8.92	0.15	[135]	2020
MISS-PAF-1	90	16.5	0.18	[136]	2020
AO-OpNpNc fibers	90	15.42	0.17	[137]	2020

Reference

1. Pettersen EF, Goddard TD, Huang CC *et al.* UCSF Chimera--a visualization system for exploratory research and analysis. *J Comput Chem* 2004; **25**: 1605-1612.
2. Anandakrishnan R, Aguilar B, Onufriev AV. H++ 3.0: automating pK prediction and the preparation of biomolecular structures for atomistic molecular modeling and simulations. *Nucleic Acids Res* 2012; **40**: W537-541.
3. Rey J, Murail S, de Vries S *et al.* PEP-FOLD4: a pH-dependent force field for peptide structure prediction in aqueous solution. *Nucleic Acids Res* 2023; **51**: W432-W437.
4. G RL. RDKit: A software suite for cheminformatics, computational chemistry, and predictive modeling. *Greg Landrum* 2013; **8**: 5281.
5. Hou XB, Du JT, Zhang J *et al.* How to Improve Docking Accuracy of AutoDock4.2: A Case Study Using Different Electrostatic Potentials. *J Chem Inf Model* 2013; **53**: 188-200.
6. Yu QH, Cui OY, Ma YJ *et al.* Bifunctional DSUP-OP fiber with high adsorption selectivity and antibiofouling activity for uranium extraction from seawater. *J Water Process Eng* 2025; **69**: 106718.
7. Ye XX, Liu J, Chen XY *et al.* Effective uranium extraction from seawater through immobilization of conjugated microporous polymers on collagen fiber membrane. *Chem Eng Sci* 2024; **295**: 120160.
8. Zhang C, Wang ZY, Ma RC *et al.* Overcoming Chemical Dissociation Processes: Electrochemical Modulation of High-Affinity Binding Sites for Rapid Uranium Extraction from Seawater. *Adv Funct Mater* 2025; **35**: 2412712.
9. Zhong LZ, Feng XF, Zhang QY *et al.* An imidazole-based covalent-organic framework enabling a super-efficiency in sunlight-driven uranium extraction from seawater. *Chem Sci* 2024; **15**: 10882-10891.
10. Yang LR, Li YH, Chen DP *et al.* Efficient cooperative extraction uranium(VI) from aqueous solution and seawater by a novel phosphate/amidoxime chitosan-based adsorbent. *J Water Process Eng* 2024; **61**: 105197.
11. Yuan Y, Cao DD, Cui FC *et al.* High-capacity uranium extraction from seawater through constructing synergistic multiple dynamic bonds. *Nature Water* 2025. doi: 10.1038/s44221-024-00346-y
12. Yi T, Cen ZH, Ji YW *et al.* Amidoxime-based Star Polymer Adsorbent with Ultra-High Uranyl Affinity for Extremely Fast Uranium Extraction from Natural Seawater. *Adv Funct Mater* 2024; **34**: 2404220.
13. Ai JY, Feng LJ, Zhang JC *et al.* Hierarchically self-supporting porous ultrathin films with aligned photothermal nanosheets for ultrafast uranium extraction from seawater. *Chem Eng J* 2024; **498**: 155754.
14. Liu T, Zhao JT, Qiao QT *et al.* Engineering shrinkage resistance of nano-structured hydrogels in seawater for fast uranium capture. *Chem Eng J* 2024; **496**: 153832.
15. Zuo LH, Li FL, Xu ZZ *et al.* Constructing yeast-modified polypropylene amidoxime membrane toward highly-selective adsorption of U(VI) from seawater. *J*

Mol Liq 2024; **414**: 126076.
16. Yu Y, Liu JY, Liu Q *et al.* High-Performance Polyamidoxime Porous Membrane Prepared by the In Situ Modification/Nonsolvent-Induced Phase Separation Strategy for Uranium Extraction from Seawater. *ACS Appl Mater Inter* 2024; **16**: 49778-49789.
17. Huang ZX, Li WY, Xu SY *et al.* A novel sponge-like composite biosorbent fabricated by sodium alginate and polyethyleneimine for uranium(VI) extraction from seawater. *Int J Biol Macromol* 2024; **279**: 135004.
18. Tuo K, Li J, Li Y *et al.* Phytic Acid Functionalized Hierarchical Porous Metal-Organic Framework Microspheres for Efficient Extraction of Uranium from Seawater. *Small* 2024; 2407272.
19. Zhang C, Li HH, Cao DD *et al.* Preparation of meso-porous aromatic frameworks for rapid ion extraction from high salt and corrosion environments. *J Mater Chem A* 2024; **12**: 17270-17276.
20. Zhao L, Wang SY, Wang G *et al.* Phosphorus Nitride Imide Nanotubes for Uranium Capture from Seawater. *ACS Nano* 2024; **18**: 11804-11812.
21. Tuo K, Li J, Li Y *et al.* Construction of hierarchical porous and polydopamine/salicylaldoxime functionalized zeolitic imidazolate framework-8 controlled etching for uranium adsorption. *Mater Horiz* 2024; **11**: 3364-3374.
22. He Q, Peng JH, Wang YM *et al.* Hollow spherical nano-traps using pillararene-based polymer for efficient uranium extraction from seawater. *Chem Commun* 2024; **60**: 9574-9577.
23. Yang JJ, Geng NB, Li Y *et al.* Wax-Casted Macroporous Polyamidoxime Hydrogel Particles Encapsulated in Alginate-Polyacrylic Acid Beads for Highly Efficient Uranium Capture from Seawater. *Adv Funct Mater* 2024; 2418340.
24. Li X, Liu Q, Chen SS *et al.* Anti-biofouling amidoxime-quaternized polyethyleneimine hemp fiber for efficient uranium extraction from seawater. *Chem Eng J* 2024; **497**: 154563.
25. Yu Y, Liu JY, Chen SS *et al.* Bioinspired electrostatic layer-by-layer assembly membranes constructed based on mild strategy for uranium extraction from seawater. *Chem Eng J* 2024; **486**: 149783.
26. Li DG, Chen Z, Zhang FQ *et al.* Nano-tentacled interconnected channels organic gel for rapid uranium extraction from seawater. *J Hazard Mater* 2024; **480**: 135784.
27. Qiang TT, Wang Y, Ren LF *et al.* Preparation of double network antibacterial hydrogel modified by hydroxamic acid gelatin/polyacrylamide and high efficiency uranium extraction from seawater. *J Environ Chem Eng* 2024; **12**: 114180.
28. Cao M, Luo GS, Peng Q *et al.* Poly(amidoxime)/polyzwitterionic semi-interpenetrating network hydrogel with robust salt-shrinkage resistance for enhanced uranium extraction from seawater. *Chem Eng J* 2024; **481**: 148536.
29. Yang L, Sun Y, Sun Y *et al.* Anti-Biofouling Polyzwitterion–Poly(amidoxime) Composite Hydrogel for Highly Enhanced Uranium Extraction from Seawater. *Gels* 2024; **10**: 603.
30. Huang C, Ma L, Mao CK *et al.* Constructing amidoxime adsorption sites on the core-shell structured natural silk protein for uranium capture. *Int J Biol Macromol*

2024; **267**: 131608.
31. Li N, Yuan SD, Su RD *et al.* Selective and antibacterial zinc phosphonate framework system for superior marine uranium harvesting. *Desalination* 2024; **586**: 117860.
32. Yao YY, Liao J, Xu X *et al.* Hydrazide and amidoxime dual functional membranes for uranium extraction from seawater. *J Mater Chem A* 2024; **12**: 10528-10538.
33. Wang Y, Zhang Y, Liu XL *et al.* Design of amidoximized hydroxyapatite for extracting uranium from seawater. *Radiat Phys Chem* 2024; **217**: 111512.
34. Qin SL, Sha JN, Yang PP *et al.* Graphene oxide/graphitic carbon nitride/polyamide oxime nanofibers for adsorption and photocatalytic reduction of uranium from seawater. *Inorganic Chemistry Frontiers* 2024; **11**: 6156-6167.
35. Huang Y, Zou SF, Li ZY *et al.* Tough polyamidoxime-nanocellulose supramolecular composite hydrogels for effective uranium extraction from seawater. *Polymer* 2024; **298**: 126895.
36. Cheng M, Liu YQ, Jiang H *et al.* Hollow multi-shelled structure engineering of organosilica for efficient and selective uranium extraction from seawater. *Desalination* 2024; **583**: 117729.
37. Li LY, Li H, Lin MZ *et al.* Zwitterionic functionalized chitosan with dual-antifouling for selective uranium extraction. *Sep Purif Technol* 2025; **354**: 128913.
38. Chen DY, Sun MF, Zhao XY *et al.* High-efficiency and economical uranium extraction from seawater with easily prepared supramolecular complexes. *J Colloid Interf Sci* 2024; **668**: 343-351.
39. Zhang YS, Wang YC, Dong ZM *et al.* Boosting uranium extraction from Seawater by micro-redox reactors anchored in a seaweed-like adsorbent. *Nat Commun* 2024; **15** : 9124.
40. Wang M, Feng LJ, Luo GS *et al.* Ultrafast extraction of uranium from seawater using photosensitized biohybrid system with bioinspired cascaded strategy. *J Hazard Mater* 2023; **445**: 130620.
41. Hao MJ, Xie Y,H Liu XL *et al.* Modulating Uranium Extraction Performance of Multivariate Covalent Organic Frameworks through Donor–Acceptor Linkers and Amidoxime Nanotraps. *JACS Au* 2023; **3**: 239-251.
42. Huang C, Fu MT, Ma L *et al.* Ultrafast strategy to self-assemble polyamidoxime PVA nanofibers with solar-induced synergy for enhancing uranium extraction from seawater. *Chem Eng J* 2023; **474**: 145718.
43. Fu MT, Huang C, Ma L *et al.* Solar enhanced uranium extraction from seawater with the efficient strategy of MXene loaded nano-porous polyamidoxime membrane. *Sep Purif Technol* 2024; **332**: 125803.
44. Wang Y, Cao M, Peng Q *et al.* Polyamidoxime-loaded biochar sphere with high water permeability for fast and effective recovery of uranium from seawater. *J Water Process Eng* 2023; **55**:104205.
45. Li T, Lin X, Zhang Z *et al.* Photothermal-Enhanced Uranium Extraction from Seawater: A Biomass Solar Thermal Collector with 3D Ion-Transport Networks. *Adv*

Funct Mater 2023; **33**: 2212819.
46. Wang Y, Jiang YY, Zhang Y *et al.* Construction of amidoxime-functionalized magnetic hydroxyapatite with enhanced uranium extraction performance from aqueous solution and seawater. *Chemosphere* 2023;**343**: 140257.
47. Xie YH, Wu Y, Liu XL *et al.* Rational design of cooperative chelating sites on covalent organic frameworks for highly selective uranium extraction from seawater. *Cell Rep Phy Sci* 2023;**4**: 101220.
48. Chen DY, Zhao XY, Jing XF *et al.* Bio-inspired functionalization of electrospun nanofibers with anti-biofouling property for efficient uranium extraction from seawater. *Chem Eng J* 2023;**445**: 142844.
49. Ou MR, Li WY,Huang ZX *et al.* Highly efficient extraction of uranium(VI) from seawater by polyamidoxime/polyethyleneimine sponge. *Sep Purif Technol* 2024;**331**: 125721.
50. Ahmad M, Ren JQ, Naik MM *et al.* New and simple synthetic strategy for two‐dimensional ultra‐microporous aromatic framework for selective uranium capture in liquid. *AIChE Journal* 2023;**69**: e18143.
51. Zhang X, Li DG, Cui C *et al.* Alginate-based supermacroporous hydrogels fabricated by cryo-polymerization for uranium extraction from seawater. *Polym Chem* 2023;**14**: 2902-2915.
52. Shan TH, Ma X, Li H *et al.* Plant-derived hybrid coatings as adsorption layers for uranium adsorption from seawater with high performance. *J Membrane Sci* 2023;**675**: 121547.
53. Yang JJ, Li Y, Tian T *et al.* Novel mesocellular silica foam supported poly(amidoxime-ethyleneimine) network for fast and highly efficient uranium extraction from seawater. *Chem Eng J* 2023;**465**: 142952.
54. Cao M, Peng Q, Wang Y *et al.* High-efficiency uranium extraction from seawater by low-cost natural protein hydrogel. *Int J Biol Macromol* 2023;**242**: 124792.
55. Cao M, Wang Y, Feng LJ *et al.* Ion-imprinted nanocellulose aerogel with comprehensive optimized performance for uranium extraction from seawater. *Chem Eng J* 2023;**475**: 146048.
56. Wu JK, Shi N, Li N *et al.* Dual-Ligand ZIF-8 Bearing the Cyano Group for Efficient and Selective Uranium Capture from Seawater. *ACS Appl Mater Inter* 2023;**15**: 46952–4696.
57. Chen DY, Zhao XY, Shi MS *et al.* Enhanced and selective uranium extraction onto electrospun nanofibers by regulating the functional groups and photothermal conversion performance. *Chem Eng J* 2024;**480**: 148108.
58. Pan ZH, Zhao L, Cai LR *et al.* Speeding up the selective extraction of uranium through in situ formed nano-pockets. *J Mater Chem A* 2023;**11**: 15437-15443.
59. Liu T, Gu AP, Wei T *et al.* Ligand‐Assistant Iced Photocatalytic Reduction to Synthesize Atomically Dispersed Cu Implanted Metal‐Organic Frameworks for Photo‐Enhanced Uranium Extraction from Seawater. *Small* 2023;**19**: 2208002.
60. Maurya A, Marvaniya K, Dobariya P *et al.* Biomimetic Helical Hydrogen Bonded Organic Framework Membranes for Efficient Uranium Recovery from Seawater. *Small* 2023;**20**: 2306824.

61. Xu YC, Zhu JH, Zhang HS *et al.* Biomimetic porous cellular foam with space thermal domains for efficient uranium extraction from seawater. *J Mater Chem A* 2023;**11**: 11264-11271.
62. Yu FT, Li CY, Li WR *et al.* Π‐Skeleton Tailoring of Olefin‐Linked Covalent Organic Frameworks Achieving Low Exciton Binding Energy for Photo‐Enhanced Uranium Extraction from Seawater. *Adv Funct Mater* 2023;**34**: 2307230.
63. Li H, Sun J, Qin SL *et al.* Zwitterion Functionalized Graphene Oxide/Polyacrylamide/Polyacrylic Acid Hydrogels with Photothermal Conversion and Antibacterial Properties for Highly Efficient Uranium Extraction from Seawater. *Adv Funct Mater* 2023;**33**: 2301773.
64. More YD, Mollick S, Saurabh S *et al.* Nanotrap Grafted Anionic MOF for Superior Uranium Extraction from Seawater. *Small* 2023;**20**: 2302014.
65. Xiao GP, Shi S, Zhao PQ *et al.* Directional dendritic gels constructed by binder-regulated freeze casting for enhanced uranium extraction from seawater. *Sep Purif Technol* 2023;**320**:124139.
66. Song YC, Tan HH, Qin SL *et al.* Assembly of a core–shell MOF with stability into Polyacrylamide hydrogel for boosting extraction of uranium from seawater. *Nano Res* 2023;**17**:3398-3406.
67. Wang Y, Lin ZW, Yu J *et al.* Biofouling-resistant polyamidoxime-based natural hierarchical porous bamboo strips prepared by in-situ polymerization for uranium extraction from seawater. *J Environ Chem Eng* 2023;**11**:109277.
68. Wang Y, Lin ZW, Zhu JH *et al.* Enhancing adsorption performance and selectivity for uranium by constructing biaxial adsorption sites on eco-friendly bamboo strips. *Sep Purif Technol* 2023;**315**:123727.
69. Li DG, Liao YZ, Chen Z *et al.* A 3D hierarchical porous adsorbent constructed by cryo-polymerization for ultrafast uranium harvesting from seawater. *J Mater Chem A* 2023;**11**:10384-10395.
70. Luo GS, Ma Y, Cao M *et al.* Salt-shrinkage resistant poly(amidoxime) adsorbent for improved extraction of uranium from seawater. *Chem Eng J* 2023;**464**:142569.
71. Lin K, Sun WY, Feng LJ *et al.* Kelp inspired bio-hydrogel with high antibiofouling activity and super-toughness for ultrafast uranium extraction from seawater. *Chem Eng J* 2022; **430**: 133121.
72. Chen L, Sun Y, Wang JW *et al.* A wood-mimetic porous MXene/gelatin hydrogel for electric field/sunlight bi-enhanced uranium adsorption. *E-Polymers* 2022;**22**: 468-477.
73. Ma DS, Xin X, Li ZW *et al.* Nanoemulsion assembly toward vaterite mesoporous CaCO3 for high-efficient uranium extraction from seawater. *J Hazard Mater* 2022;**432**:128695.
74. Yang LR, Luo X, Yan L *et al.* Efficient selective adsorption of uranium using a novel eco-friendly chitosan-grafted adenosine 5′-monophosphate foam. *Carbohyd Polym* 2022;**285**: 119157.
75. Feng LJ, Wang H, Feng TT *et al.* In Situ Synthesis of Uranyl‐Imprinted Nanocage for Selective Uranium Recovery from Seawater. *Angew Chem Int Ed* 2022;**61**: e202101015.

76. Yang LS, Xiao HY, Qian YC *et al.* Bioinspired hierarchical porous membrane for efficient uranium extraction from seawater. *Nat Sustain* 2021;**5**: 71-80.
77. Jiao GJ, Ma JL, Zhang JQ *et al.* Porous and biofouling-resistant amidoxime-based hybrid hydrogel with excellent interfacial compatibility for high-performance recovery of uranium from seawater. *Sep Purif Technol* 2022;**287**:120571.
78. Shi S, Wu R, Meng SL *et al.* High-strength and anti-biofouling nanofiber membranes for enhanced uranium recovery from seawater and wastewater. *J Hazard Mater* 2022;**436**: 128983.
79. Wang H, Xu TH, heng BH *et al.* Cuttlefish ink loaded polyamidoxime adsorbent with excellent photothermal conversion and antibacterial activity for highly efficient uranium capture from natural seawater. *J Hazard Mater* 2022;**433**:128789.
80. Wang H, Zheng BH, Xu TH *et al.* Macroporous hydrogel membrane by cooperative reaming for highly efficient uranium extraction from seawater. *Sep Purif Technol* 2022;**289**: 120823.
81. Liu Tao , Zhang XB, Gu AP *et al.* In-situ grown bilayer MOF from robust wood aerogel with aligned microchannel arrays toward selective extraction of uranium from seawater. *Chem Eng J* 2022;**433**: 134346.
82. Pu YD, Qiang TT, Ren LF. Waste feather fiber based high extraction capacity bio-adsorbent for sustainable uranium extraction from seawater. *Int J Biol Macromol* 2022;**206**: 699-707.
83. Liu T, Zhang RQ, Chen MW *et al.* Vertically Aligned Polyamidoxime/Graphene Oxide Hybrid Sheets' Membrane for Ultrafast and Selective Extraction of Uranium from Seawater. *Adv Funct Mater* 2021;**32**: 2111049.
84. Li Nan, Gao P, Chen HW *et al.* Amidoxime modified Fe3O4@TiO2 particles for antibacterial and efficient uranium extraction from seawater. *Chemosphere* 2022;**287**: 132137.
85. Feng SW, Feng LJ, Wang M *et al.* Highly efficient extraction of uranium from seawater by natural marine crab carapace. *Chem Eng J* 2022;**430**: 133038.
86. Wang W, Luo Qiang , Li JY *et al.* Single-atom tungsten engineering of MOFs with biomimetic antibiofilm activity toward robust uranium extraction from seawater. *Chem Eng J* 2022;**431**: 133483.
87. Liu T, Xie ZJ, Chen MW *et al.* Mussel-inspired dual-crosslinked polyamidoxime photothermal hydrogel with enhanced mechanical strength for highly efficient and selective uranium extraction from seawater. *Chem Eng J* 2022;**430**: 133182.
88. Sun WY, Feng LJ, Zhang JC *et al.* Amidoxime Group‐Anchored Single Cobalt Atoms for Anti‐Biofouling during Uranium Extraction from Seawater. *Adv Sci* 2022;**9**: 2105008.
89. Ratnitsai V, Wongjaikham W, Wongsawaeng D *et al.* Highly promising recycled low-density polyethylene sheet adsorbents for uranium recovery from seawater. *J Nuclr Sci Technol* 2021;**59**: 629-640.
90. Xu X, Huang CH, Wang YJ *et al.* Engineering biaxial stretching polyethylene membrane with poly(amidoxime)-nanoparticle and mesopores architecture for uranium extraction from seawater. *Chem Eng J* 2022;**430**: 133159-133159.

91. Gu HQ, Ju PH, Liu Q *et al.* Constructing an Amino-reinforced amidoxime swelling layer on a Polyacrylonitrile surface for enhanced uranium adsorption from seawater. *J Colloid Interf Sci* 2022;**610**: 1015-1026.
92. Yang H, Liu XL, Hao MJ *et al.* Functionalized Iron–Nitrogen–Carbon Electrocatalyst Provides a Reversible Electron Transfer Platform for Efficient Uranium Extraction from Seawater. *Adv Mater* 2021;**33**: 2106621.
93. Hu BW, Wang HF, Liu RR *et al.* Highly efficient U(VI) capture by amidoxime/carbon nitride composites: Evidence of EXAFS and modeling. *Chemosphere* 2021;**274**: 129743.
94. Wang N, Zhao XM, Wang JW *et al.* Accelerated Chemical Thermodynamics of Uranium Extraction from Seawater by Plant‐Mimetic Transpiration. *Adv Sci* 2021;**8**: 2102250.
95. Qin XD, Yang WT, Yang WK *et al.* Covalent modification of ZIF-90 for uranium adsorption from seawater. *Microp Mesopor Mater* 2021;**233**: 111231.
96. Cui WR, Zhang CR, Xu RH *et al.* Low Band Gap Benzoxazole‐Linked Covalent Organic Frameworks for Photo‐Enhanced Targeted Uranium Recovery. *Small* 2021;**17**: 2006882.
97. Ma L, Gao J, Huang C *et al.* UiO-66-NH-(AO) MOFs with a New Ligand BDC-NH-(CN) for Efficient Extraction of Uranium from Seawater. *ACS Appl Mater Inter* 2021;**13**: 57831-57840.
98. Wang D, Liu Z, Yue YR *et al.* Blow spinning of pre–acid-activated polyamidoxime nanofibers for efficient uranium adsorption from seawater. *Mater Today Energy* 2021;**21**: 100735.
99. Cui WR, Zhang CR, Liang RP *et al.* Covalent organic framework hydrogels for synergistic seawater desalination and uranium extraction. *J Mater Chem A* 2021;**9**: 25611-25620.
100. Wen SX, Sun Y, Liu RR *et al.* Supramolecularly Poly(amidoxime)-Loaded Macroporous Resin for Fast Uranium Recovery from Seawater and Uranium-Containing Wastewater. *ACS Appl Mater Inter* 2021;**13**: 3246–3258.
101. Cui WR, Zhang CR, Liang RP *et al.* Covalent Organic Framework Sponges for Efficient Solar Desalination and Selective Uranium Recovery. *ACS Appl Mater Inter* 2021;**13**: 31561–31568.
102. Li N, Yang L, Wang D *et al.* High-Capacity Amidoxime-Functionalized β-Cyclodextrin/Graphene Aerogel for Selective Uranium Capture. *Environ Sci Technol* 2021;**55**: 9181–9188.
103. Yuan YH, Yu QH, Cao M *et al.* Selective extraction of uranium from seawater with biofouling-resistant polymeric peptide. *Nat Sustain* 2021;**4**: 708-714.
104. Yu ZQ, Ye DP, Zhao J *et al.* Photocatalytic anti-biofouling coatings with dynamic surfaces of hybrid metal-organic framework nanofibrous mats for uranium (VI) separation from seawater. *Chem Eng J* 2021;**420**: 129691.
105. Sun Y, Liu RR, Wen SX *et al.* Antibiofouling Ultrathin Poly(amidoxime) Membrane for Enhanced U(VI) Recovery from Wastewater and Seawater. *ACS Appl Mater Inter* 2021;**13**: 21272-21285.
106. Liu RR, Wen SX, Sun Y *et al.* A nanoclay enhanced

Amidoxime-Functionalized Double-Network hydrogel for fast and massive uranium recovery from seawater. *Chem Eng J* 2021;422: 130060.
107. Cheng G, Zhang AR, Zhao ZW *et al.* Extremely stable amidoxime functionalized covalent organic frameworks for uranium extraction from seawater with high efficiency and selectivity. *Sci Bull* 2021;**66**: 1994-2001.
108. Wu YD, Cui WR, Zhang CR *et al.* Regenerable, anti-biofouling covalent organic frameworks for monitoring and extraction of uranium from seawater. *Environl Chem Lett* 2021;**19**: 1847–1856.
109. Liu Tao, Zhang XB, Wang H *et al.* Photothermal enhancement of uranium capture from seawater by monolithic MOF-bonded carbon sponge. *Chem Eng J* 2021;**412**: 128700.
110. He NN, Li H, Li LY *et al.* Polyguanidine-modified adsorbent to enhance marine applicability for uranium recovery from seawater. *J Hazard Mater* 2021;**416**:126192.
111. Wang Ying, Lin ZW, Liu Q *et al.* Simple one-step synthesis of woven amidoximated natural material bamboo strips for uranium extraction from seawater. *Chem Eng J* 2021;**425**: 131538.
112. Chen MW, Liu T, Zhang XB *et al.* Photoinduced Enhancement of Uranium Extraction from Seawater by MOF/Black Phosphorus Quantum Dots Heterojunction Anchored on Cellulose Nanofiber Aerogel. *Adv Funct Mater* 2021;**31**: 2100106.
113. uan YH, Guo X, Feng LJ *et al.* Charge balanced anti-adhesive polyacrylamidoxime hydrogel membrane for enhancing uranium extraction from seawater. *Chem Eng J* 2021;**431**: 127878.
114. Song YP, Zhu CJ, Sun Q *et al.* Nanospace Decoration with Uranyl-Specific “Hooks” for Selective Uranium Extraction from Seawater with Ultrahigh Enrichment Index. *ACS Cent Sci* 2021;7: 1650-1656.
115. Das S, Wang ZY, Brown S *et al.* Strategies toward the Synthesis of Advanced Functional Sorbent Performance for Uranium Uptake from Seawater. *Ind Eng Chem Res* 2021;**60**: 15037-15044.
116. Sun Q, Song YP, Aguila B *et al.* Spatial Engineering Direct Cooperativity between Binding Sites for Uranium Sequestration. *Adv Sci* 2020;**8**: 2001573.
117. Guo XJ, Yang HC, Liu Q *et al.* A chitosan-graphene oxide/ZIF foam with anti-biofouling ability for uranium recovery from seawater. *Chem Eng J* 2020;**382**: 122850.
118. Yu QH, Yuan YH, Feng LJ *et al.* Spidroin‐Inspired, High‐Strength, Loofah‐Shaped Protein Fiber for Capturing Uranium from Seawater. *Angew Chem Int Ed* 2020;**59**: 15997-16001.
119. Yang Sen, Cao Y, Wang T *et al.* Positively charged conjugated microporous polymers with antibiofouling activity for ultrafast and highly selective uranium extraction from seawater. *Environl Res* 2020;**183**: 109214.
120. Yuan YH, Liu TT, Xiao JX *et al.* DNA nano-pocket for ultra-selective uranyl extraction from seawater. *Nat Commun* 2020;**11**: 5708.
121. Cui WR, Li FF, Xu RH *et al.* Regenerable Covalent Organic Frameworks for Photo‐enhanced Uranium Adsorption from Seawater. *Angew Chem Int Ed* 2020;**59**: 17684-17690.

122. Yan BJ, Ma CX, Gao JX *et al.* An Ion‐Crosslinked Supramolecular Hydrogel for Ultrahigh and Fast Uranium Recovery from Seawater. *Adv Mater* 2020;**32**:1906615.
123. Yuan YH, Feng SW, Feng LJ *et al.* A Bio‐inspired Nano‐pocket Spatial Structure for Targeting Uranyl Capture. *Angewe Chem Int Ed* 2020;**59**: 4262-4268.
124. Shi S, Li BC, Qian YX *et al.* A simple and universal strategy to construct robust and anti-biofouling amidoxime aerogels for enhanced uranium extraction from seawater. *Chem Eng J* 2020;**397**: 125337.
125. Xu HB, Bai ZY, Zhang ML *et al.* Water-locking molecule-assisted fabrication of nature-inspired Mg(OH)2 for highly efficient and economical uranium capture. *Dalton T* 2020;**49**: 7535-7545.
126. Li Z, Yu ZQ, Wu YD *et al.* Self-sterilizing diblock polycation-enhanced polyamidoxime shape-stable blow-spun nanofibers for high-performance uranium capture from seawater. *Chem Eng J* 2020;**390**: 124648.
127. Shi S, Qian YX, Mei PP *et al.* Robust flexible poly(amidoxime) porous network membranes for highly efficient uranium extraction from seawater. *Nano Energy* 2020;**71**: 104629.
128. Bai ZY, Liu Q, Zhang HS *et al.* Anti-Biofouling and Water—Stable Balanced Charged Metal Organic Framework-Based Polyelectrolyte Hydrogels for Extracting Uranium from Seawater. *ACS Appl Mater Inter* 2020;**12**: 18012–18022.
129. Li WT, Liu YY, Bai Y *et al.* Anchoring ZIF-67 particles on amidoximerized polyacrylonitrile fibers for radionuclide sequestration in wastewater and seawater. *J Hazard Mater* 2020;**395**: 122692.
130. Li Hao, He NN, Cheng C *et al.* Antimicrobial polymer contained adsorbent: A promising candidate with remarkable anti-biofouling ability and durability for enhanced uranium extraction from seawater. *Chem Eng J* 2020;**388**: 124273.
131. Gao JX, Yuan YH, Yu QH *et al.* Bio-inspired antibacterial cellulose paper–poly(amidoxime) composite hydrogel for highly efficient uranium(vi) capture from seawater. *Chem Commun* 2020;**56**: 3935-3938.
132. Yuan YH, Niu BY, Yu QH *et al.* Photoinduced Multiple Effects to Enhance Uranium Extraction from Natural Seawater by Black Phosphorus Nanosheets. *Angew Chem Int Ed* 2020;**59**: 1220-1227.
133. Xu X, Yue YR, Cai D *et al.* Aqueous Solution Blow Spinning of Seawater‐Stable Polyamidoxime Nanofibers from Water‐Soluble Precursor for Uranium Extraction from Seawater. *Small Methods* 2020;**4**: 2000558.
134. Pan HB, Wai CM, Kuo LJ *et al.* A highly efficient uranium grabber derived from acrylic fiber for extracting uranium from seawater. *Dalton T* 2020; **49**(9): 2803-2810.
135. Li ZN, Meng QH, Yang YJ *et al.* Constructing amidoxime-modified porous adsorbents with open architecture for cost-effective and efficient uranium extraction. *Chem Sci* 2020;**11**: 4747-4752.
136. Wang ZY, Meng QH, Ma RC *et al.* Constructing an Ion Pathway for Uranium Extraction from Seawater. *Chem* 2020;**6**: 1683-1691.
137. Xu X, Xu L, Ao JX *et al.* Ultrahigh and economical uranium extraction from

seawaterviainterconnected open-pore architecture poly(amidoxime) fiber. *J Mater Chem A* 2020;**8**: 22032-22044.
